# MYBL2-Driven Transcriptional Programs Link Replication Stress and Error-prone DNA Repair With Genomic Instability in Lung Adenocarcinoma

**DOI:** 10.3389/fonc.2020.585551

**Published:** 2021-01-08

**Authors:** Benjamin B. Morris, Nolan A. Wages, Patrick A. Grant, P. Todd Stukenberg, Ryan D. Gentzler, Richard D. Hall, Wallace L. Akerley, Thomas K. Varghese, Susanne M. Arnold, Terence M. Williams, Vincenzo Coppola, David R. Jones, David T. Auble, Marty W. Mayo

**Affiliations:** ^1^ Department of Biochemistry & Molecular Genetics, University of Virginia, Charlottesville, VA, United States; ^2^ Department of Pathology, University of Virginia, Charlottesville, VA, United States; ^3^ Department of Public Health Sciences, University of Virginia, Charlottesville, VA, United States; ^4^ Department of Biomedical Science, Florida Atlantic University, Boca Raton, FL, United States; ^5^ Division of Medical Oncology, Department of Internal Medicine, Hematology/Oncology, University of Virginia Health System, Charlottesville, VA, United States; ^6^ Department of Medical Oncology, Department of Internal Medicine, Huntsman Cancer Institute, Salt Lake City, UT, United States; ^7^ Division of Thoracic Surgery, Department of Surgery, University of Utah, Salt Lake City, UT, United States; ^8^ Department of Internal Medicine, Division of Medical Oncology, Markey Cancer Center, Lexington, KY, United States; ^9^ Department of Radiation Oncology, The Ohio State University Comprehensive Cancer Center, Columbus, OH, United States; ^10^ Department of Cancer Biology and Genetics, The Ohio State University Comprehensive Cancer Center, Columbus, OH, United States; ^11^ Department of Thoracic Surgery, Memorial Sloan-Kettering Cancer Center, New York, NY, United States

**Keywords:** MYBL2, error-prone DNA repair, homologous recombination (HR), lung adenocarcinoma, microhomology mediated-end joining repair (MMEJ)

## Abstract

It has long been recognized that defects in cell cycle checkpoint and DNA repair pathways give rise to genomic instability, tumor heterogeneity, and metastasis. Despite this knowledge, the transcription factor-mediated gene expression programs that enable survival and proliferation in the face of enormous replication stress and DNA damage have remained elusive. Using robust omics data from two independent studies, we provide evidence that a large cohort of lung adenocarcinomas exhibit significant genome instability and overexpress the DNA damage responsive transcription factor MYB proto-oncogene like 2 (MYBL2). Across two studies, elevated *MYBL2* expression was a robust marker of poor overall survival and disease-free survival outcomes, regardless of disease stage. Clinically, elevated *MYBL2* expression identified patients with aggressive early onset disease, increased lymph node involvement, and increased incidence of distant metastases. Analysis of genomic sequencing data demonstrated that *MYBL2* High lung adenocarcinomas had elevated somatic mutation burden, widespread chromosomal alterations, and alterations in single-strand DNA break repair pathways. In this study, we provide evidence that impaired single-strand break repair, combined with a loss of cell cycle regulators TP53 and RB1, give rise to MYBL2-mediated transcriptional programs. Omics data supports a model wherein tumors with significant genomic instability upregulate MYBL2 to drive genes that control replication stress responses, promote error-prone DNA repair, and antagonize faithful homologous recombination repair. Our study supports the use of checkpoint kinase 1 (CHK1) pharmacological inhibitors, in targeted *MYBL2* High patient cohorts, as a future therapy to improve lung adenocarcinoma patient outcomes.

## Introduction

Genomic instability, a hallmark of cancer, is a key driver of disease evolution and progression (1). Several groups have shown that genomic instability promotes metastasis and poor patient outcomes, regardless of tumor type (2–4). Decades of research demonstrates that double-strand DNA breaks produce chromosomal translocations and widespread genome instability (5). Cells contain two major pathways that repair double-strand DNA breaks, non-homologous end joining (NHEJ) and high-fidelity homologous recombination (HR). However, cancer cells commonly carry deleterious mutations that significantly decrease cellular capacity for faithful DNA repair (5, 6). Notably, mutations in homologous recombination effectors Breast Cancer Susceptibility Type 1 (BRCA1) and 2 (BRCA2) significantly compromise HR repair (7). Additionally, it is now understood that mutations in BRCA-associated genes or genes that govern replication fork protection also decrease HR and increase reliance on error-prone DNA repair mechanisms (6, 8). As a result of this reliance, these tumors demonstrate significant genomic instability as evidenced by increased telomeric alterations, large scale chromosomal transitions, and loss of heterozygosity events (9–12). While studies have linked mutant DNA repair effectors with genomic instability, a significant focus of the clinical community has been to identify drivers of decreased HR capacity and genomic instability phenotypes in tumors that contain wildtype effector genes. One of the most robust markers of defective HR in cancer is elevated mRNA expression of *RAD51*, an ATPase central to HR repair (13–16). At sites of stalled DNA replication, RAD51 protects single-strand DNA and facilitates recruitment of BRCA1/2 (7, 8, 17). Additionally, RAD51, and its homologs, directly participate in HR repair by facilitating strand-invasion of homologous DNA sequences. Not surprisingly, several studies have demonstrated that many cancers, including carcinomas of the lung, upregulate RAD51 to compensate for defective HR pathways (13–16).

Lung cancer is the leading cause of cancer related deaths worldwide. Histologically, approximately 80% of lung cancers are non-small cell lung cancers (NSCLC). Lung adenocarcinoma is the most prevalent subtype of NSCLC and has a five-year overall survival rate of less than 18% (18, 19). The poor survival rates observed in lung adenocarcinoma are directly linked to the frequent development of distant metastases to the liver, bone, and brain. Like many other carcinomas, lung adenocarcinomas exhibit significant genome instability without displaying mutations in HR genes (20). While it is recognized that lung adenocarcinomas can exhibit genomic markers of defective HR, the molecular programs governing these phenotypes are not understood (20). Identifying the pro-tumor programs that drive genomic instability in treatment naïve lung adenocarcinomas will provide novel opportunities to improve patient outcomes.

In this study, we provide evidence that lung adenocarcinomas displaying ineffective HR overexpress the DNA-damage responsive transcription factor MYB proto-oncogene like 2 (MYBL2) (*MYBL2* High) (21). Functionally, MYBL2 binds to the MUVB transcriptional complex composed of LIN9, LIN37, LIN52, LIN54, and RBBP4 to upregulate genes in late G1/S and early G2 cell cycle phases (22–24). While dysregulated *MYBL2* expression has been linked to genomic instability and poor outcomes in multiple carcinomas, including lung, the pro-tumor transcriptional programs regulated by MYBL2 have remained elusive (22). Here, we describe a MYBL2-driven transcriptional program that promotes error-prone double-strand break repair, genomic instability, and poor patient outcomes in lung adenocarcinoma. Comprehensive molecular profiling of *MYBL2* High lung adenocarcinomas provide evidence that this transcriptional program arises due to defects in single-strand DNA break repair and TP53/RB1 tumor suppressors, rather than mutations in HR effectors.

## Materials and Methods

### Study Design

We sought to identify drivers of novel genomic instability phenotypes in lung adenocarcinomas with wildtype HR effectors using omics data available from The Cancer Genome Atlas (TCGA) and the Oncology Research Information Exchange Network (ORIEN) consortium. TCGA Firehose Legacy (Lung Adenocarcinoma) data was obtained from cBioPortal (25). Differential expression analyses were conducted using cBioPortal’s Group Comparison tool for normalized TCGA RNA-sequencing and proteomic (RPPA) data. Genomic data and DNA repair metrics were made available for 515 TCGA Firehose Legacy samples by Knijenburg et al. (20, Supplementary File “TCGA_DDR_Data_Resources.xlsx” ). Catalogue of Somatic Mutations in Cancer (COSMIC) signature data was provided for 515 TCGA Firehose Legacy samples by mSignatureDB (http://tardis.cgu.edu/tw/msignaturedb/) (26). TCGA *MYBL2* High or Low samples lacking specific data points (mutational burden, repair proficiency score (RPS) values, etc.) were excluded on a test by test basis for statistical evaluation purposes. Exact patient numbers are reported for both *MYBL2* High and Low cohorts in each figure legend. Access to a novel lung adenocarcinoma cohort was provided by the ORIEN consortium. Data in the July 2019 ORIEN private cBioPortal instance was analyzed in this study.

### Patient Stratification

**Figure 1:**
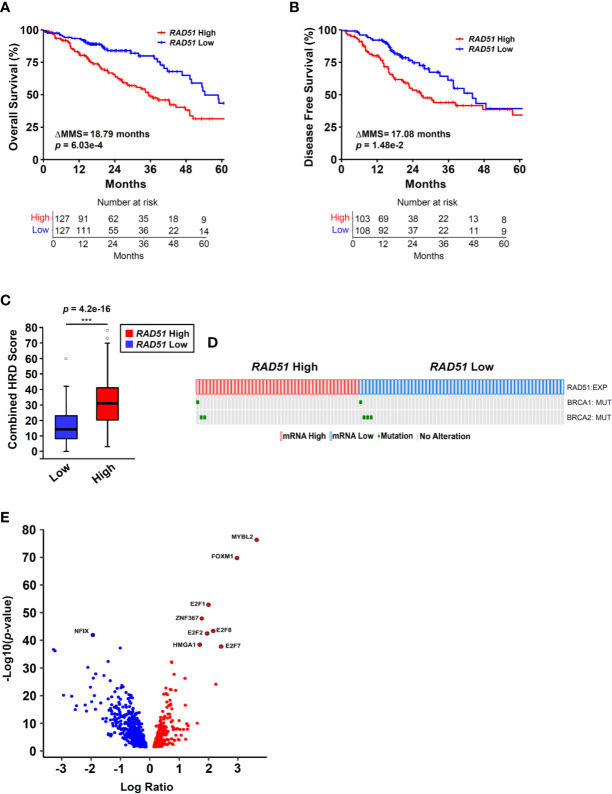
RAD51 overexpression links MYBL2 with genomic instability in BRCA wildtype lung adenocarcinoma. **(A)** Kaplan-Meier analyses reveal that patients with *RAD51* High tumors have significantly worse OS outcomes, compared to patients with *RAD51* Low tumors (log-rank *p* = 6.03e–4, ΔMMS = 18.79 months). **(B)** Kaplan-Meier analyses reveal that *RAD51* High tumors are more likely to recur, compared to *RAD51* Low tumors (log-rank *p* = 1.48e–2, ΔMMS = 17.08 months). **(C)** *RAD51* High tumors (N = 122) exhibit elevated Combined HRD scores, compared to *RAD51* Low (N = 121) (T-test, two-sided) (20). Boxplot data is displayed as a five-number summary corresponding to data minimum, first quartile, median, third quartile, and maximum. **(D)** Oncoprint profiling reveals BRCA1 and BRCA2 mutations are rare in both *RAD51* High (N = 46) and *RAD51* Low (N = 58) cohorts (27). **(E)** MYBL2 is the most differentially expressed transcription factor upregulated in *RAD51* High tumors (Log Ratio = 3.64, *q* = 1.68e–74) (28).

**Figure 2:**
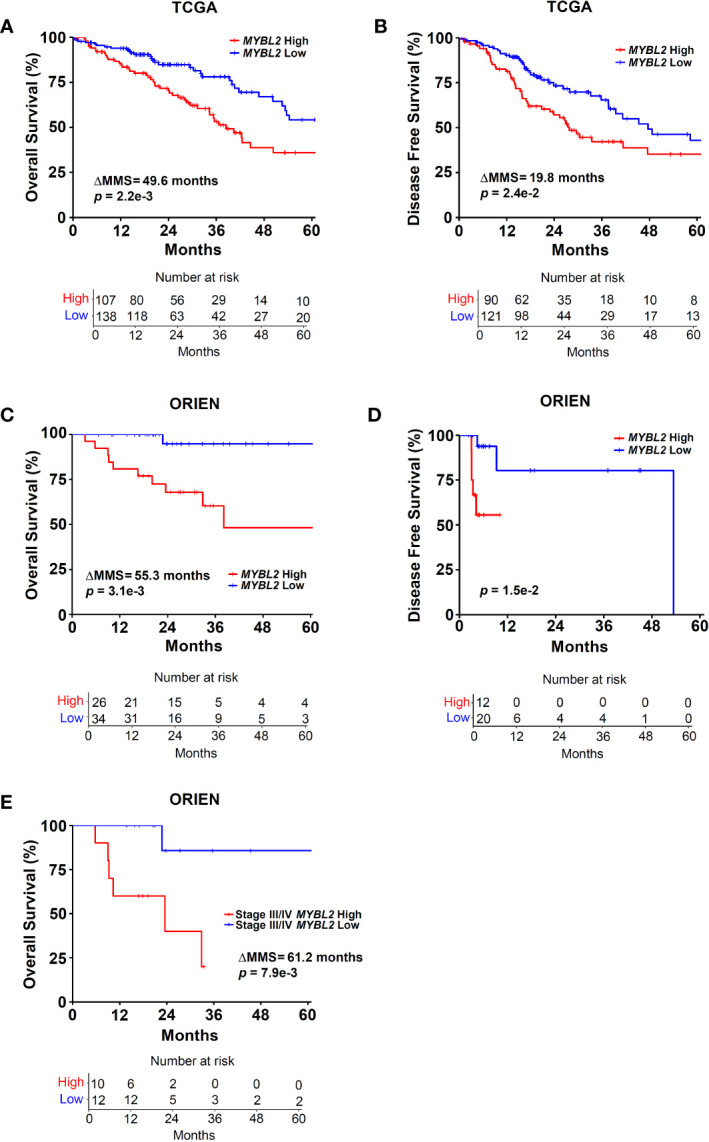
Elevated *MYBL2* mRNA expression is a strong predictor of poor outcomes in lung adenocarcinoma. **(A)** Kaplan-Meier analyses demonstrate that patients with *MYBL2* High tumors have significantly poorer OS rates, compared to patients with *MYBL2* Low tumors (log-rank *p* = 2.2e–3, ΔMMS = 49.6 months). **(B)** Kaplan-Meier analyses indicate that *MYBL2* High tumors are significantly more likely to recur, compared to *MYBL2* Low tumors (log-rank *p* = 2.4e–2, ΔMMS = 19.8 months). **(C)** Kaplan-Meier survival analyses confirm that patients with *MYBL2* High have significantly poorer OS outcomes, compared to *MYBL2* Low when validated in an independent cohort provided by ORIEN (log-rank *p* = 3.1e–3, ΔMMS = 55.3 months). **(D)** Kaplan-Meier analyses confirm that ORIEN *MYBL2* High tumors are significantly more likely to recur, compared to ORIEN *MYBL2* Low tumors (log-rank *p* = 1.5e–2). **(E)** OS Kaplan-Meier analysis reveals that *MYBL2* is a strong marker of patient outcomes in Stage III/IV disease (log-rank *p* = 7.9e–3, ΔMMS = 61.2 months).

TCGA samples with RNA-sequencing data were stratified into *RAD51* High and Low cohorts using a quartile-based approach; the top 25% of samples expressing *RAD51* were called *RAD51* High and the bottom 25% of samples were called *RAD51* Low (**Figure 1**). For MYBL2 analyses, TCGA samples with RNA-sequencing data were stratified into *MYBL2* High and Low cohorts using a modified quartile-based approach (**Figure 2**). Here, the top 21% of TCGA lung adenocarcinomas expressing *MYBL2* were called *MYBL2* High and the bottom 27% of samples were called *MYBL2* Low. These cutoffs were chosen after exploratory analyses demonstrated that they produced significantly more robust biological signals as measured by false discovery rate (FDR) values following RNA-sequencing (RNA-seq) and RPPA differential expression analyses. This fits well given, when stratifying on expression of a transcription factor, more stringent upper thresholds maximize transcription-factor specific biologic signal. To validate our findings, we applied the same cutoffs when analyzing a novel, independent lung adenocarcinoma cohort provided by ORIEN. Here, ORIEN lung adenocarcinomas with RNA-seq data were stratified into *MYBL2* High and Low cohorts using our modified quartile-based approach; the top 21% of ORIEN samples expressing *MYBL2* were called *MYBL2* High and the bottom 27% were called *MYBL2* Low.

### Survival Analyses

The Kaplan-Meier product limit estimator was used to estimate time-to-event distributions for OS and DFS. The log-rank test was used to test for differences in time-to-event distributions with a two-sided test. For both TCGA and ORIEN, OS refers to the time between initial diagnosis and time of death. DFS refers to the time between initial therapy and disease progression or death. Patients who did not experience an event or were lost to follow-up were considered censored at the time of last follow-up/contact. Cox proportional hazard models were used to assess the prognostic value of individual risk factors for TCGA patient OS and DFS outcomes. For both OS and DFS Cox proportional hazards models, patient smoking history and tumor (T) stage variables were dichotomized ahead of analyses. Kaplan-Meier survival analyses and Cox proportional hazards modeling were conducted using survminer and survival R packages (29).

### Clinical Endpoint Analyses

Clinical data accompanying TCGA and ORIEN tumors were analyzed for several specific endpoints. For TCGA tumors, we investigated potential differences in overall survival (OS), disease-free survival (DFS), tumor (T) stage, lymph node (N) involvement, metastatic (M) disease codes, age at diagnosis, patient smoking history, and tumor size when comparing *MYBL2* High and Low cohorts. Tumor size was manually extracted from digital pathology reports accompanying TCGA tumors. For ORIEN tumors, we analyzed potential differences in OS, DFS, disease-stage, and metastatic disease sites between *MYBL2* High and Low cohorts.

### Gene Set Enrichment Analysis

WEB-based GEne SeT AnaLysis Toolkit (WebGestalt) was used to analyze a pre-ranked list of differentially expressed genes between TCGA *MYBL2* High and *MYBL2* Low tumors (30). The pre-ranking metric used was as follows: (*sign of Log Ratio*) * (-log_10_(*p* - value)).

### Chromatin Immunoprecipitation Sequencing Analysis

Replicate data sets were analyzed for MYBL2 ChIP-Seq reads and broad peaks (GEO : GSM1010876). ChIP-seq data for histone specific modifications were downloaded for H3K27Ac (GEO : GSM733743) and H3K4me3 (GEO : GSM733737). ChIP-seq reads were aligned to the human hg19 reference genome and visualized using the Integrated Genome Viewer tool (31). To identify candidate MYBL2-regulated DNA damage response genes, a list of all MYBL2 ChIP-seq broad peaks was merged with a list of genes whose expression was significantly altered when comparing both *MYBL2* High and *MYBL2* Low TCGA (N = 248) and ORIEN (N = 79) patient cohorts (**Figure 5A**). For key genes involved in replication fork protection (RFP), microhomology-mediated end joining (MMEJ), and HJ-rejection, DNA sequences corresponding to the MYBL2 ChIP-Seq broad peaks were analyzed for LIN54 *cis*-elements using MEME suite (32). Identified *cis*-elements were analyzed using JASPAR (33). The highest affinity LIN54 DNA binding site identified in each promoter is reported in **Figure 5D**.

### Cell Culture

Lung adenocarcinoma cell lines A549, NCI-H23, NCI-H1568, and NCI-H1651 were obtained from American Type Culture Collection (ATCC, Manassas, VA). A549 and H23 cells were maintained in RPMI-1640 medium supplemented with 10% fetal bovine serum (FBS), penicillin, and streptomycin. H1651 were cultured in DMEM:F12 medium, as recommended by ATCC, with the following modifications. DMEM:F12 culture media was supplemented with EGF (10 ng/mL final concentration), 1% FBS, and penicillin/streptomycin. H1568 cells were cultured using the same DMEM:F12 supplemented media as described for H1651.

### Western Blotting and Antibodies

Cell extracts were isolated using RIPA lysis method and westerns were performed as described previously (34). Primary antibodies were used at 1:1000 according to manufacturer specifications. Primary antibodies include: MYBL2 (Millipore #MABE886), CHK1 (Novus Biologicals #NB100-464), αTubulin (Sigma-Aldrich #T9026), γH2AX pS139 (Cell Signaling #9718), and H2AX (Cell Signaling #7631). Secondary antibodies conjugated to HRP include anti-rabbit IgG (Cell Signaling #7074) and anti-mouse IgG (Cell Signaling #7076). Secondary antibodies were used at 1:2500. Densitometric analyses were performed on autoradiographs and fold change relative to tubulin loading control was calculated using NIH ImageJ 1.46r software.

### Small Molecule Inhibitor and PrestoBlue HS Cell Viability Assays

NSCLC cells were seeded onto 24 well culture plates at a density of 1.3 x 10^5^ cells per well (~60% confluency) on Day 0. On Day 1, inhibitors were added to culture media and mixed thoroughly. On Day 3, culture media was aspirated, cells were washed with 1 mL of PBS, and subsequently incubated with fresh media and PrestoBlue HS cell viability dye (ThermoFisher P50200); PrestoBlue HS cell viability dye was added at a 1:10 (volume:volume) ratio according to manufacturer instructions. PrestoBlue HS dye was also incubated with cell-free, media only controls to account for background signal from culture media. After PrestoBlue HS addition, culture plates were incubated at 37°C for two hours. Following incubation, PrestoBlue HS fluorescence signal was quantified using a SpectraMax M2 microplate reader. Resulting signal was background corrected and is reported as a ratio of 560/590 nm fluorescence.

### Statistical Analyses

Statistical tests used throughout this study are indicated within figure legends. For all boxplots, data is displayed as minimum, first quartile, median, third quartile, and maximum. For all bar graphs, data is presented as mean +/- standard deviation. For all analyses, *p* and *q* (False Discovery Rate, FDR) values < 0.05 were considered statistically significant.

## Results

### Elevated RAD51 mRNA Expression Links MYBL2 with Genomic Instability in BRCA Wildtype Lung Adenocarcinoma

To identify transcriptional programs associated with genomic instability and poor outcomes in lung adenocarcinoma, we stratified lung adenocarcinomas from The Cancer Genome Atlas (TCGA, TCGA Firehose Legacy, N = 517) on *RAD51* mRNA expression using a quartile-based approach (*Materials and Methods*). Since elevated *RAD51* gene expression is commonly associated with cancers with defective HR repair pathways, we stratified tumors based on *RAD51* mRNA expression to identify lung adenocarcinomas with elevated genomic instability (13–16). Kaplan-Meier analyses confirmed that patients with *RAD51* High lung adenocarcinomas had significantly worse OS and DFS outcomes, compared to patients with *RAD51* Low lung adenocarcinomas (**Figures 1A, B**). Carcinomas with defective HR commonly feature widespread chromosomal alterations, characteristic of genomic instability (9–12). Using data generated by the TCGA PanCancer Atlas consortium, we found that *RAD51* High tumors had significantly elevated combined homologous recombination deficiency (combined HRD) scores, compared to *RAD51* Low (**Figure 1C**) (20). The combined HRD metric represents the sum of all telomeric allelic imbalances (NtAI), large scale transitions (LST, >10 Mb), and loss of heterozygosity (LOH, >15 Mb) events observed in individual tumors (20, 35). High combined HRD scores reflect widespread chromosomal alterations and are frequently observed in tumors with defective HR. Mutations in BRCA1 and BRCA2 are canonical drivers of decreased HR capacity and genomic instability phenotypes (5, 6, 8). Using whole exome sequencing data accompanying TCGA tumors, we profiled *RAD51* High tumors to assess for the presence of BRCA1 or BRCA2 mutations. Surprisingly, we found that BRCA1/2 mutations were rare in both *RAD51* High and *RAD51* Low cohorts (**Figure 1D**). Taken together, these data indicated that *RAD51* overexpression successfully identified BRCA1/2 wildtype tumors with significant genomic instability and poor survival outcomes (**Figures 1C, D**).

Next, we sought to identify the transcription factor(s) driving *RAD51* High lung adenocarcinomas. To do this, we systematically screened all known human transcription factors against a list of significantly differentially expressed (*q* < 0.05) genes between *RAD51* High and *RAD51* Low tumors (28). MYB proto-oncogene like 2 (MYBL2) was the highest differentially expressed transcription factor upregulated in *RAD51* High lung adenocarcinomas (**Figure 1E**). Functionally, MYBL2 governs gene expression in G1/S and early G2 cell cycle phases by binding to the large multi-subunit MUVB complex composed of LIN9, LIN37, LIN52, LIN54, and RBBP4 (22–24). Other transcription factors that functionally cooperate with MYBL2 to drive transcription (E2F1, E2F2, E2F7, E2F8) or are directly regulated by MYBL2 (FOXM1) were also significantly upregulated (**Figure 1E**).

### Elevated *MYBL2* mRNA Expression Predicts Poor Patient Outcomes

Given the association between *RAD51* and *MYBL2* expression, we examined whether stratifying lung adenocarcinomas on *MYBL2* mRNA expression alone could predict OS and DFS outcomes. TCGA lung adenocarcinomas were stratified into *MYBL2* High and *MYBL2* Low cohorts using a modified quartile-based method (*Materials and Methods*). Subsequent Kaplan-Meier analyses revealed that patients with *MYBL2* High lung adenocarcinomas had significantly worse OS rates, compared to patients with *MYBL2* Low lung adenocarcinomas (ΔMMS = 49.6 months, log-rank *p* = 2.2e–3) (**Figure 2A**). Additionally, we found that *MYBL2* High tumors were more likely to recur when compared to *MYBL2* Low (ΔMMS = 19.8 months, log-rank *p* = 2.42e–2) (**Figure 2B**). For both OS and DFS analyses, the *MYBL2* Low cohort reached median survival beyond 60 months. Subsequent survival analyses confirmed that overexpression of MYBL2 outperformed both E2F1 and FOXM1 transcription factors in identifying lung adenocarcinoma patients with poor outcomes (**Figure 2**, **Figure S1**). Key proteins that work in concert with MYBL2 to regulate transcription, namely E2F family transcription factors and the MUVB complex, were selectively upregulated in *MYBL2* High, suggesting that MYBL2 actively regulated the behavior of these tumors (**Figure S2**).

To validate our findings, we repeated patient stratification and survival analyses using a novel lung adenocarcinoma cohort from the ORIEN consortium (N = 165) (*Materials and Methods*). In this independent cohort, patients with *MYBL2* High tumors again had significantly worse OS and DFS rates (OS: ΔMMS = 55.3 months, log-rank *p* = 3.1e–3; DFS: log-rank *p* = 1.5e–2) (**Figures 2C, D**). As with the TCGA cohort, ORIEN *MYBL2* Low patients reached median OS beyond 60 months. Importantly, a separate analysis of only Stage III and IV lung adenocarcinoma confirmed that patients with *MYBL2* High tumors had significantly worse OS outcomes compared to patients with *MYBL2* Low tumors (log-rank *p* = 7.9e–3, **Figure 2E**). Taken together, these data identify elevated *MYBL2* mRNA expression as a robust predictor of poor outcomes in lung adenocarcinoma, regardless of disease stage.

### *MYBL2* High Disease Is Associated With Adverse Clinical Characteristics and Genetic Alterations

When reviewing clinical endpoints accompanying *MYBL2* High and Low tumors, we found that *MYBL2* High disease had several distinguishing characteristics. First, *MYBL2* High patients were significantly younger at diagnosis in both TCGA (*p* = 1.5e–3) and ORIEN (*p* = 5.7e–4) cohorts (**Table 1**). TCGA *MYBL2* High tumors were significantly larger at diagnosis (*p* = 0.016) and presented with increased regional lymph node involvement (**Table 1**). ORIEN patients with *MYBL2* High tumors displayed an increased prevalence of distant metastases, with increased dissemination to the brain, liver, and kidney (**Figure S3A**). We also found that 75% of TCGA *MYBL2* High patients were current or recently reformed smokers (<15 years) at diagnosis, while 64% of *MYBL2* Low patients were either lifelong non-smokers or reformed for >15 years (Chi-squared *p* = 8.65e–10, **Table 1**). Analysis of commonly altered oncogenes and tumor suppressors revealed that TCGA *MYBL2* High tumors had coincident alterations in the RAS, TP53, and RB1 pathways (**Figure S3B**). More specifically, *MYBL2* High tumors had more alterations that activated the RAS pathway and disrupted TP53 and RB1 tumor suppressor pathways. Consistent with previous findings in other carcinomas, inactivating alterations in TP53 were highly enriched in *MYBL2* High lung adenocarcinomas (36); 76% of *MYBL2* High tumors contained TP53 mutations, compared to only 19% of *MYBL2* Low (*q* = 4.2e–4, one-sided Fisher Exact test, Benjamini-Hochberg corrected) (**Figure S3B**). Collectively, we found that this *MYBL2* High phenotype was associated with early onset disease, presentation of larger tumors, increased regional lymph node involvement, increased prevalence of distant metastases, TP53 mutations, and recent cessation of or continued cigarette smoking.

**Table 1:** Baseline Clinical Characteristics for TCGA and ORIEN MYBL2 cohorts.

TCGA				ORIEN			
	*MYBL2* High(N = 108)	*MYBL2* Low(N = 140)	*p*-value		*MYBL2* High(N = 34)	*MYBL2* Low(N = 45)	*p*-value
**Average Age at Diagnosis, years** **(range)**	64 (40–88)	68 (41–87)	1.5e–3*	**Average Age at Diagnosis, years** **(range)**	62 (44–79)	69 (45-83)	5.7e–4*
**Male**	56.5 (61)	40.7 (57)		**Male**	44.1 (15)	40 (18)	
**Tumor Stage**				**Disease Stage**			
**1**	25 (27)	42.1 (59)		**I**	14.7 (5)	24.4 (11)	
**2**	63 (68)	45 (63)		**II**	20.6 (7)	22.2 (10)	
**3**	10.2 (11)	7.9 (11)		**III**	11.76 (4)	20 (9)	
**4**	1.9 (2)	4.3 (6)		**IV**	17.6 (6)	8.9 (4)	
**TX**	0 (0)	0.7 (1)		**NA**	35.3 (12)	24.4 (11)	
**Metastasis** **Code**				**Metastatic Disease**			
**M0**	63 (68)	67.9 (95)		**None**	38.2 (13)	60 (27)	
**M1**	6.5 (7)	2.1 (3)		**Regional**	11.8 (4)	17.8 (8)	
**M1a**	0.9 (1)	0 (0)		**Distant**	29.4 (10)	13.3 (6)	
**M1b**	0.9 (1)	0.7 (1)		**Regional & Distant**	11.8 (4)	4.4 (2)	
**MX**	27.8 (30)	27.9 (39)		**NA**	8.8 (3)	4.4 (2)	
**NA**	0.9 (1)	1.4 (2)	
**Lymph Node Involvement**			
**N0**	63.9 (69)	78.6 (110)	
**N1**	24.1 (26)	10 (14)	
**N2**	12 (13)	7.1 (10)	
**N3**	0 (0)	0 (0)	
**NX**	0 (0)	3.6 (5)	
**NA**	0 (0)	0.7 (1)	
**Patient Smoking History**			8.65e-10**
**Life-long** **Non-smoker**	7.4 (8)	22.6 (32)	
**Current smoker**	36.1 (39)	8.6 (12)	
**Reformed >15 years**	14.8 (16)	41.4 (58)	
**Reformed ≤15 years**	38.9 (42)	23.6 (33)	
**Reformed, not specified**	0.9 (1)	0.7 (1)	
**Not annotated**	1.9 (2)	2.9 (4)	
**Average** **Tumor Size,** **Largest Dimension (cm)**	4.2	3.9	0.016***

All data are presented as percentage (number of patients) unless otherwise specified. Life-long non-smokers are defined as patients who have smoked <100 cigarettes in their lifetime. The current smoker designation includes both daily and non-daily smokers. *Student’s t-test. **Chi-squared Test. ***Wilcoxon test.

### Cox Proportional Hazards Modeling Demonstrated That MYBL2 Is a Robust Prognostic Marker for Both Overall Survival and Disease-Free Survival Outcomes

Given that elevated *MYBL2* expression correlated with several clinical characteristics linked to poor patient outcomes, we performed multivariate Cox hazard modeling to assess the prognostic value of *MYBL2*, while adjusting for other risk factors. Using clinical characteristics from **Table 1**, we built multivariate survival models for TCGA OS and DFS outcomes based on *MYBL2* expression, patient smoking history, patient age at diagnosis, and tumor (T) stage (**Table 2**, *Materials and Methods*). For this analysis, tumor (T) stage was selected because it captures both local invasion and tumor size datapoints (37). When analyzing our multivariate OS model results, we found that high *MYBL2* expression (HR = 2.50, *p* = 2.45e–4), age at diagnosis (HR = 1.03, *p* = 8.51e–3), and high tumor (T) stage (HR = 1.69, *p* = 1.36e–3) were all significantly associated with diminished OS. Patient smoking history did not have a significant effect on OS (HR = 1.09, *p* = 0.404). For DFS outcomes, we also found that high *MYBL2* expression (HR = 2.00, *p* = 3.72e–3), age at diagnosis (HR = 1.03, *p* = 4.95e–3), and high tumor (T) stage (HR = 1.63, *p* = 1.63e–3) were significantly associated with diminished DFS. As with OS, patient smoking history did not have a significant effect on DFS outcomes (HR = 0.157, *p* = 0.152). Together, this data confirmed that *MYBL2* expression is an important prognostic variable for both OS and DFS patient outcomes, after adjusting for other key clinical factors such as age at diagnosis, tumor (T) stage, and patient smoking history.

**Table 2:** TCGA Cox Multivariate Survival Analysis. 95% CI, 95% confidence interval.

TCGA Overall Survival				TCGA Disease-Free Survival			
Risk Factor	Regression	Hazard Ratio (95% CI)	*p*-value	Risk Factor	Regression	Hazard Ratio (95% CI)	*p*-value
	Coefficient				Coefficient		
***MYBL2* expression**	0.916	2.50 (1.53–4.08)	2.45e–4*	***MYBL2* expression**	0.695	2.00 (1.25–3.21)	3.72e–3*
**Patient smoking history**	0.082	1.09 (0.87–1.35)	0.464	**Patient smoking history**	0.157	1.17 (0.94–1.45)	0.152
**Age at Diagnosis**	0.034	1.03 (1.01–1.06)	8.51e–3*	**Age at Diagnosis**	0.034	1.03 (1.01–1.06)	4.95e–3*
**Tumor Stage**	0.523	1.69 (1.23–2.33)	1.36e–3*	**Tumor Stage**	0.489	1.63 (1.20–2.21)	1.63e–3*
Concordance = 0.677, Standard error = 0.037				Concordance = 0.684, Standard error = 0.036			
Likelihood Ratio Test *p* = 5.0e–5*				Likelihood Ratio Test *p* = 3.0e–4*			

*Statistically significant.

### *MYBL2* High Lung Adenocarcinoma Demonstrate Significant Genomic Instability and Defective HR Repair Despite Containing Wildtype BRCA

**Figure 3:**
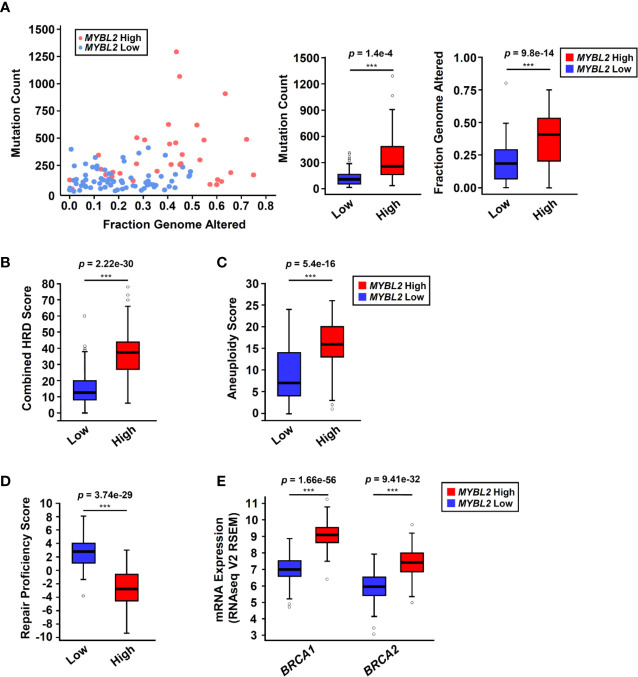
*MYBL2* High tumors exhibit significant genome instability and inefficient homologous recombination. **(A)** *MYBL2* High tumors (N = 33) had significantly greater mutation burden compared to *MYBL2* Low tumors (N = 72) (*p* = 1.4e-4, T-test, two-sided). *MYBL2* High tumors (N = 109) also had significantly greater fractions of their genomes altered, compared to *MYBL2* Low (N = 139) (*p* = 9.8e–14, T-test, two-sided). **(B)** *MYBL2* High lung adenocarcinomas (N = 104) display elevated combined homologous recombination deficiency (combined HRD) scores, compared to *MYBL2* Low (N = 134) (*p* = 2.22e–30, T-test, two-sided). **(C)** *MYBL2* High (N = 104) tumors exhibit increased aneuploidy scores, compared to *MYBL2* Low (N = 134) (*p* = 5.4e–16, T-test, two-sided). Combined HRD-scores and aneuploidy scores were calculated and reported by the TCGA PanCancer Atlas consortium (20). **(D)** *MYBL2* High (N = 77) lung adenocarcinomas have significantly lower repair proficiency score (RPS) values when compared to *MYBL2* Low (N = 136) (*p* = 3.74e–29, T-test, two-sided). **(E)** *MYBL2* High lung adenocarcinoma overexpressed *BRCA1* (*p* = 1.66e–56, T-test, two-sided) and *BRCA2* (*p* = 9.41e–32, T-test, two-sided), compared to *MYBL2* Low lung adenocarcinoma. **(A–E)** Boxplot data is displayed as a five-number summary corresponding to data minimum, first quartile, median, third quartile, and maximum.

Analysis of TCGA sequencing data demonstrated that *MYBL2* High tumors had significantly higher somatic mutation load (*p* = 1.4e–4) and increased genomic alterations (*p* = 9.8e–14), compared to *MYBL2* Low (**Figure 3A**). As expected, we found that *MYBL2* High lung adenocarcinomas had significantly higher combined HRD scores (*p* = 2.22e–30) (**Figure 3B**). *MYBL2* High tumors also had significantly higher numbers of chromosome arm-level gains and losses, compared to *MYBL2* Low (Aneuploidy Score, *p* = 5.4e–16) (**Figure 3C**). Collectively, these data indicated that *MYBL2* High tumors demonstrated marked genomic instability.

A hallmark of genomic instability in BRCA mutant tumors is decreased cellular capacity for HR repair (5, 6, 8). In 2014, Pitroda and colleagues developed a metric, termed the repair proficiency score (RPS), that quantifies the ability of cells to undergo HR. Using this metric, low RPS values reflect decreased HR capacity (16). Here we found that *MYBL2* High tumors exhibited significantly lower RPS values, indicating that these tumors do not effectively undergo HR (**Figure 3D**) (16). Analysis of whole exome sequencing data revealed that the incidence of mutations in BRCA1 and BRCA2 genes was low in both *MYBL2* High and Low cohorts (BRCA1: 0% in *MYBL2* High, 1.39% in *MYBL2* Low; BRCA2: 12.12% in *MYBL2* High, 4.17% in *MYBL2* Low) (**Figure S4**). Importantly, BRCA1/2 mutations were not enriched in *MYBL2* High tumors, compared to *MYBL2* Low (BRCA1: q = 0.486, BRCA2: q = 0.382; one-sided Fisher Exact test, Benjamini-Hochberg corrected). Moreover, we found that *BRCA1* and *BRCA2* transcripts were significantly overexpressed in *MYBL2* High tumors (**Figure 3E**). Taken together, these data confirmed that *MYBL2* High lung adenocarcinomas exhibited a novel genomic instability phenotype with inefficient HR in the presence of highly expressed, wildtype BRCA1/2.

**Figure 4:**
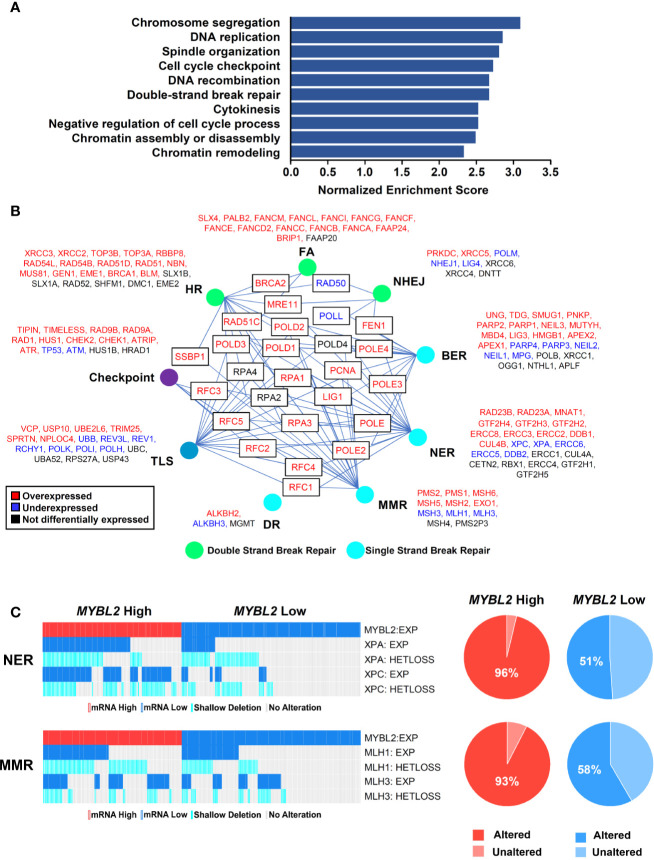
*MYBL2* High tumors display an elevated DNA damage response and contain defective single-strand break repair pathways. **(A)** Gene set enrichment analysis (GSEA) revealed that *MYBL2* High tumors significantly overexpressed genes involved in the biologic processes shown. **(B)** *MYBL2* High lung adenocarcinoma displayed transcriptional alterations in DNA damage response (DDR) pathways. HR, Homologous recombination; FA, Fanconi anemia; NHEJ, non-homologous end joining; BER, base excision repair; NER, nucleotide excision repair; MMR, mismatch repair; DR, direct repair; TLS, translesion synthesis. **(C)** Oncoprint profiling of key mismatch repair (MMR) and nucleotide excision repair (NER) genes for *MYBL2* High and *MYBL2* Low tumors (27).

To investigate potential mechanisms linking MYBL2 with genome instability, we analyzed a list of genes differentially expressed between TCGA *MYBL2* High and Low tumors using GSEA (30). GSEA showed that *MYBL2* High tumors significantly overexpressed genes directing DNA replication, DNA repair, cell cycle, cytokinesis, and chromatin organization (**Figure 4A**). Given the widespread genome instability observed in *MYBL2* High tumors, we found it intriguing that DNA repair pathways were among the most upregulated. Next, we systematically mapped all differentially expressed DNA damage response (DDR) genes to identify any potential defects in DNA damage sensing (checkpoint), single-strand break repair, or double-strand break repair pathways (**Figure 4B**) (38). We found that *MYBL2* High tumors lacked deleterious alterations in checkpoint, HR, or Fanconi Anemia (FA) repair pathways. While *ATM* transcript was significantly under-expressed in *MYBL2* High tumors, ATM was not significantly suppressed at the protein level (**Supplementary Data Sheet 2**). Although translesion synthesis (TLS), non-homologous end joining (NHEJ), direct repair (DR), and base-excision repair (BER) pathways had significantly downregulated genes, it was unlikely that these were major contributors to *MYBL2* High pathogenesis due to potential compensation from other intact pathway effectors. Interestingly, we found that nucleotide excision repair (NER) was significantly impaired in *MYBL2* High tumors due to the loss of irreplaceable effectors *XPA* and *XPC* (**Figure 4C**). We also found mismatch repair (MMR) to be impaired in *MYBL2* High due to the loss of *MLH1* and *MLH3* (**Figure 4C**). While defective NER and MMR pathways could partially account for increased mutation burden (**Figure 3A**), these alterations did not explain the widespread chromosomal alterations observed in *MYBL2* High tumors (**Figures 3A–C**).

### *MYBL2* High Lung Adenocarcinomas Express Genes That Drive Replication Stress Responses and Error-Prone DNA Repair

**Figure 5:**
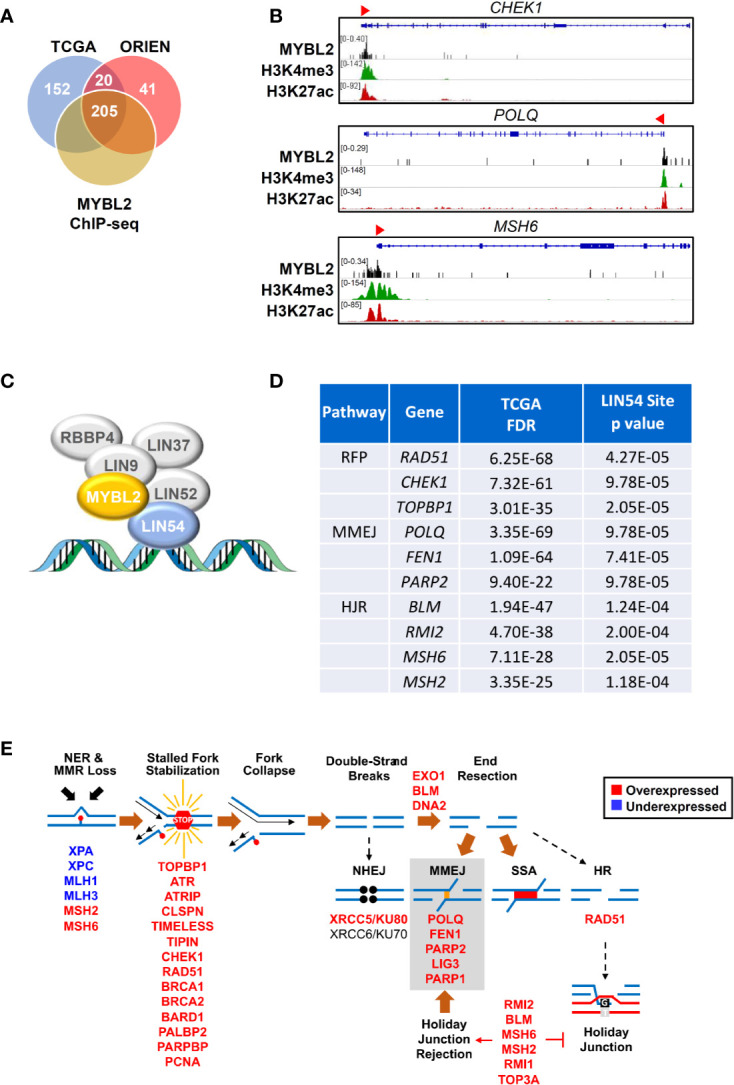
MYBL2 drives expression of genes that antagonize homologous recombination and promote error prone DNA repair. **(A)** Analysis of chromatin immunoprecipitation sequencing (ChIP-seq) data revealed MYBL2 localization at the promoters of 205 DNA damage response genes significantly altered in both TCGA and ORIEN *MYBL2* High tumors. **(B)** Representative Integrated Genome Viewer (IGV) snapshots of ChIP-seq enrichment peaks showing MYBL2 and active transcription histone mark co-occupancy at promoters of key DNA damage response genes. **(C)** MYBL2 bound to the LIN54:MUVB complex drives transcription. **(D)** Key replication fork protection (RFP), microhomology-mediated end joining (MMEJ), and HJ-rejection genes contained high affinity LIN54 sites in their promoters. **(E)** Omics data support a model wherein MYBL2 drives expression of genes that antagonize homologous recombination and promote error-prone repair. NHEJ: non-homologous end joining, MMEJ: microhomology-mediated end joining, SSA: single-strand annealing, HR: homologous recombination. Solid brown arrows indicate dominant repair pathway fate. Solid arrow (→) and inhibition (┴) symbols indicate activation or antagonization, respectively. Hashed black arrows indicate inefficient pathways.

Given the low RPS values in *MYBL2* High lung adenocarcinomas, we hypothesized that MYBL2 directly upregulated genes that antagonized HR and promoted error-prone DNA repair. To test this hypothesis, we identified DDR genes whose expression was significantly altered in both TCGA and ORIEN *MYBL2* High cohorts (*Materials and Methods*, **Figure 5A**). DDR genes were considered direct MYBL2 targets if they contained both MYBL2 ChIP-seq enrichment peaks and high-affinity LIN54 *cis*-elements in their promoters (**Figure 5C**). Approximately 91% (205/225) of the DNA damage response genes altered in both TCGA and ORIEN cohorts contained MYBL2 ChIP-seq enrichment peaks at or near transcriptional start sites (**Figure 5A**, **Supplementary Data Sheet 3**). Screen shots from the Integrated Genome Viewer tool demonstrates MYBL2 ChIP-seq enrichment peaks upstream of *CHEK1*, *POLQ*, and *MSH6* promoters (**Figure 5B**). Importantly, MYBL2 ChIP-seq enrichment peaks at transcriptional start sites correlated with histone modifications (H3K4me3, H3K27Ac) commonly associated with active transcription (39).

In examining these 205 candidate MYBL2-regulated genes, we found a concerted upregulation of genes involved in three main processes: sensing and protection of stalled replication forks, error-prone microhomology-mediated end joining (MMEJ) repair, and inhibition of HR through Holliday junction rejection (HJ-rejection) (**Figures 5C–E**). Critical enzymes in each of these pathways contained MYBL2 ChIP-seq enrichment peaks and high-affinity LIN54 *cis*-elements in their promoters, indicating that these genes were bonafide targets of the MYBL2:LIN54 transcriptional complex (**Figure 5D**).

As shown previously, *MYBL2* High tumors exhibited defective NER and MMR pathways (**Figure 4C**). Impaired NER and MMR pathways cause widespread replication stress, which was evident given the significant overexpression of genes that sense and stabilize stalled replication forks (**Figure 5E**) (40). Inability to repair DNA lesions at stalled replication forks promote replication fork collapse and double-strand break formation (40, 41). *MYBL2* High tumors upregulated enzymes that promote end-resection of double-strand DNA breaks, namely *EXO1*, *BLM*, and *DNA2* (**Figure 5E**). This cohort also selectively upregulated genes driving error-prone MMEJ with the rate-limiting enzyme *POLQ* being one of the most significantly upregulated DDR genes (**Figures 5D–E**). Equally important, *MYBL2* High tumors overexpressed genes composing the BLM-RMI complex that governs HJ-rejection (40). The BLM-RMI complex blocks HR when unrepaired mismatched nucleotides are present in a sister chromosome template sequence that is being used for HR repair (40, 42). Without intact MMR pathways due to the loss of *MLH1* and *MLH3*, HJ-rejection antagonizes faithful HR and promotes error-prone MMEJ repair (**Figure 5E**) (40). Collectively, these data are consistent with a mechanism wherein MYBL2 drives a previously undefined phenotype by upregulating negative regulators of HR as well as key effectors that enable MMEJ repair.

### Omics Data Support a MYBL2-Centric Genomic Instability Model

**Figure 6:**
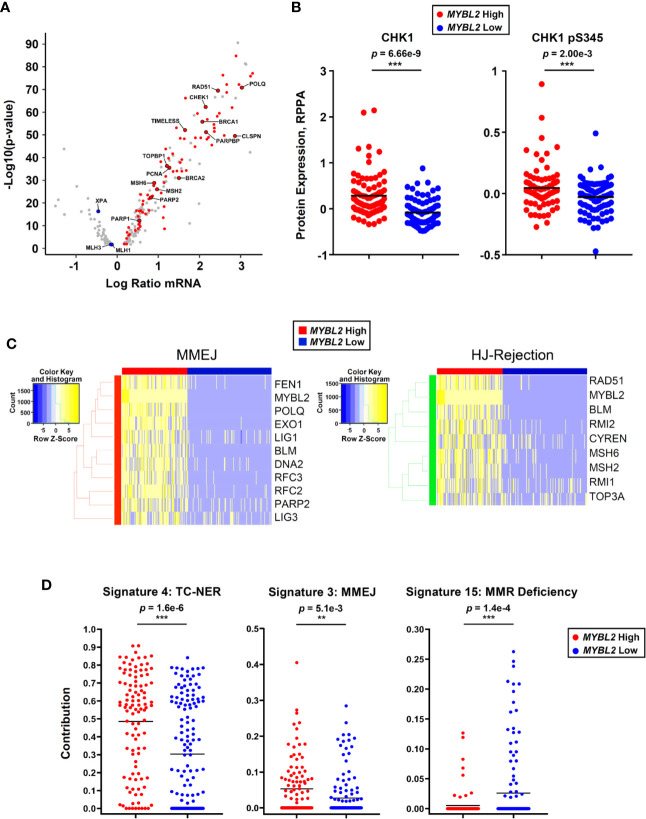
*MYBL2* High lung adenocarcinoma exhibit significant replication stress, concerted upregulation of MMEJ and HJ-rejection genes, and genomic evidence of error-prone repair. **(A)** *MYBL2* High tumors overexpressed genes involved in replication stress response, ATR/CHK1 checkpoint signaling, and microhomology-mediated end joining (MMEJ) repair. **(B)** Reverse phase protein array (RPPA) data revealed elevated CHK1 (T-test, two-sided) and phospho-CHK1 (pS345, T-test, two-sided) protein in *MYBL2* High tumors (N = 82), compared to *MYBL2* Low (N = 91). **(C)** TCGA *MYBL2* High tumors selectively overexpressed genes driving MMEJ and HJ-rejection pathways. **(D)** COSMIC mutational signature data revealed transcription-coupled nucleotide excision repair (TC-NER, T-test, two-sided) and microhomology mediated end-joining (MMEJ, T-test, two-sided) accounted for significantly greater proportions of mutations in *MYBL2* High LUAD (N = 107) (26, 43). Mismatch repair deficiency (MMR Deficiency, T-test, two-sided) accounts for significantly more mutations in *MYBL2* Low tumors (N = 140) (43). Lines denote mean mutation contribution per tumor.

Since **Figure 5E** was developed solely based on RNA-Seq and ChIP-Seq analyses, we sought additional omics evidence to support our *MYBL2* High lung adenocarcinoma model. Of the 205 DDR genes analyzed, 89 (43%) genes sense and respond to replication stress (**Figure 6A**). Many of these genes are among the highest expressed DDR genes in *MYBL2* High tumors, suggesting these tumors experience chronic replication stress. Consistent with this notion, analysis of proteomic data revealed that *MYBL2* High tumors had significantly elevated CHK1 and phospho-CHK1 protein (CHK1-S345p), indicative of a chronic, ATR-mediated intra-S checkpoint response due to replication stress (**Figure 6B**). Our model is further supported by the fact *MYBL2* High tumors selectively upregulate genes governing MMEJ and HJ-rejection mechanisms (**Figure 6C**).

As defective DNA repair results in distinct footprints observable in the cancer genome, we analyzed COSMIC mutational signature data for sequence-level evidence of error-prone DNA repair in *MYBL2* High tumors (26, 43). Of the 30 annotated COSMIC mutation signatures, only Signatures 4 and 3 accounted for significantly greater proportions of mutations in *MYBL2* High tumors (**Supplementary Table 1**) (43). Forty-eight percent of all mutations across *MYBL2* High tumors were attributed to COSMIC Signature 4 (*p* = 1.6e–6, **Figure 6D**). Signature 4 is defined by C>A transversions driven by tobacco carcinogens and errors in transcription-coupled (TC)-NER (43). This data provides sequence level evidence that *MYBL2* High tumors had significantly impaired NER due to the loss of *XPA* and *XPC* (**Figures 4C**, **5E**). Consistent with our overall model (**Figure 5E**), *MYBL2* High tumors had significantly elevated Signature 3 mutations characteristic of MMEJ repair (*p* = 5.1e–3), (**Figure 6D**) **(** 43, 44 **)**. Finally, Signature 15, which describes mutations stemming from MMR defects, accounted for more mutations in *MYBL2* Low tumors (*p* = 1.4e–4) (43). This finding fits well with our model given that *MYBL2* Low tumors fail to undergo BLM-RMI mediated HJ-rejection, which enables mismatched nucleotides to be pseudo-repaired via MMEJ (**Figure 6D**).

### The Checkpoint Kinase Inhibitor, Prexasertib, Demonstrates Effective Cytotoxic Activity *In Vitro*

**Figure 7:**
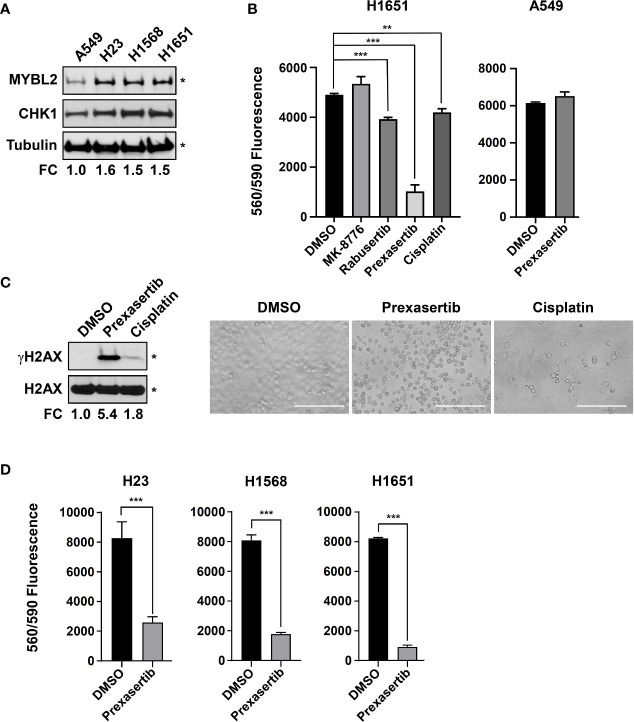
The CHK inhibitor, prexasertib, is cytotoxic to *MYBL2* High lung adenocarcinoma cells. **(A)** Western blot analysis of cellular extracts demonstrated elevated MYBL2 and CHK1 protein expression in H23, H1568, and H1651, relative to A549. **(B)** Prexasertib treatment outperforms MK-8776, rabusertib, and cisplatin. All compounds were assayed at 1 μM. Conditions were assayed as triplicate wells; similar data was obtained in an independent experiment. **(C)** Twenty-four hour treatment with prexasertib effectively induces DNA damage, as evidenced by accumulation of γH2AX, compared to cisplatin and DMSO vehicle control. Photomicrographs demonstrated the cytotoxic activity of prexasertib, compared to cisplatin and DMSO vehicle control 48 hour post-treatment. White scale bar: 200 μm (20x). **(D)** The cytotoxic activity of prexasertib (250 nM) is observed across multiple *MYBL2* High cell lines. Conditions were tested in triplicate. Data presented represents the average of triplicate wells across two combined independent experiments. **(A, C)** densitometric quantification of autoradiographs, represented as fold change (FC), demonstrated differential expression of MYBL2 protein across lung adenocarcinoma cell lines, relative to tubulin loading control (*). **(B, D)** Two-sided Student’s t-test was used to test for differences between inhibitor treated wells and DMSO vehicle controls. *p < 0.05, **p < 0.01, ***p < 0.001.

Robust transcriptomic and proteomic data demonstrate that elevated CHK1 activity is a hallmark of *MYBL2* High tumors (**Figures 6A, B**). Given that *MYBL2* High patients have significantly poorer outcomes (**Figure 2**, **Tables 1**, **2**), we explored the cytotoxic efficacy of small molecule CHK1 inhibitors in *MYBL2* High lung adenocarcinoma cells. RNA-seq data from the Cancer Cell Line Encyclopedia (CCLE) was used to identify *MYBL2* High and *MYBL2* Low cell lines. Importantly, cell lines with elevated *MYBL2* transcript showed increased MYBL2 and CHK1 protein expression by western analysis (H23, H1568, H1651), compared to *MYBL2* Low cells (A549) (**Figure 7A**). Following cell line identification, we tested three small molecule inhibitors of CHK1 for cytotoxic activity *in vitro*. At a uniform dose of 1 μM, prexasertib was the most effective cytotoxic agent, significantly outperforming MK-8776, rabusertib, and cisplatin (**Figure 7B**). Interestingly, prexasertib was not cytotoxic to *MYBL2* Low A549 cells (**Figure 7B**). Western analysis for γH2AX in H1651 cell extracts confirmed that prexasertib treatment significantly impaired repair following DNA damage, relative to cisplatin or vehicle control (**Figure 7C**). Photomicrographs of H1651-treated cells demonstrated the effectiveness of prexasertib-induced cytotoxicity, compared to cisplatin or DMSO vehicle control (**Figure 7C**). The ability of prexasertib to effectively induce cellular cytotoxicity was not cell line specific but was observed in multiple *MYBL2* High cell lines (H23, H1568, H1651) (**Figure 7D**). Collectively, our data supports the use of prexasertib, an effective CHK1 inhibitor, for targeting *MYBL2* High lung adenocarcinoma cells displaying widespread replication stress and ineffective HR repair.

### *MYBL2* High Lung Adenocarcinoma: Patient Identification

Moving forward, reliably identifying *MYBL2* High disease in the clinic is of the upmost importance. To this end, we developed an RNA-based tumor profiling panel that distinguishes *MYBL2* High lung adenocarcinomas across both TCGA and ORIEN cohorts, regardless of disease stage (**Figure 7A**). This panel consists of genes involved in cell cycle progression (*BUB1, CCNA2, CCNB1, FOXM1, MYBL2, GTSE1*), replication stress (*WDHD1, TIMELESS, CDC45, RRM2, RAD51*), error-prone DNA repair (*POLQ, EXO1*), and lung differentiation (*SFTPB*). Elevated expression of each panel gene tracked independently with significantly poorer OS and DFS outcomes when tested using TCGA data (**Supplementary Data Sheet 4**).

**Figure 8:**
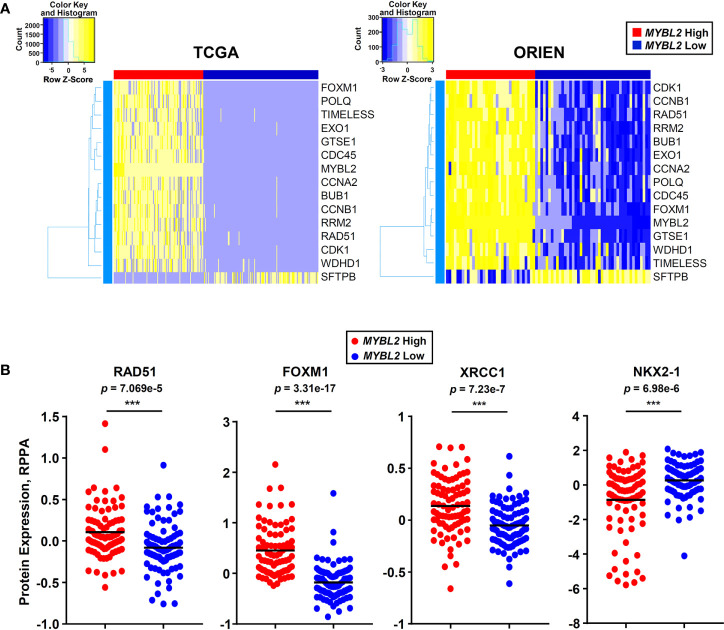
*MYBL2* High lung adenocarcinoma patient identification. **(A)** A 15 gene panel reliably identified *MYBL2* High lung adenocarcinoma across TCGA and ORIEN cohorts. **(B)** *MYBL2* High (N = 82) tumors significantly overexpress RAD51 (T-test, two-sided), XRCC1 (T-test, two-sided), and FOXM1 (T-test, two-sided) proteins, compared to *MYBL2* Low (N = 91). *MYBL2* High tumors also exhibit significantly decreased expression of NKX2-1 protein (T-test, two-sided), a key marker of lung differentiation.

In parallel to developing an RNA expression-based panel (**Figure 8A**), we also analyzed TCGA proteomic data to identify candidate immunohistochemistry (IHC) markers for *MYBL2* High disease. We found that *MYBL2* High lung adenocarcinomas significantly overexpressed DNA repair proteins that support replication fork stability and MMEJ repair (**Supplementary Data Sheet 2**). Specifically, *MYBL2* High tumors overexpressed CHK1, RAD51, and X-ray Repair Cross Complementing 1 (XRCC1), which helps recruit POLQ for MMEJ repair (**Figures 6B** and **8B**). Moreover, these tumors also overexpressed FOXM1, a direct transcriptional target of MYBL2, and underexpressed the lung differentiation homeobox transcription factor, NKX2-1 (**Figure 8B**) (22). This data suggests that a combined IHC panel detecting MYBL2, FOXM1, RAD51, CHK1, and XRCC1 could be used to reliably identify *MYBL2* High lung adenocarcinomas. Accurately identifying this cohort of patients will help tailor future therapeutic interventions, direct clinical trial design, and ultimately improve patient outcomes.

## Discussion

Across two independent studies, elevated *MYBL2* expression identified lung adenocarcinoma patients with significantly poorer OS and DFS outcomes, early onset disease, increased regional lymph node involvement, and increased prevalence of distant metastases (**Figure 2**, **Table 1**, **Figure S3A**). Importantly, Cox proportional hazards modeling demonstrated that *MYBL2* is a robust prognostic marker for both OS and DFS patient outcomes (**Table 2**). Analysis of omics data revealed that *MYBL2* High lung adenocarcinomas had significantly elevated somatic mutations and widespread chromosomal alterations characteristic of genomic instability (**Figures 3A–C**). Since increased mutations and chromosomal rearrangements are linked with tumor heterogeneity, disease recurrence, and metastasis, we sought to understand the MYBL2-driven programs promoting disease progression in lung adenocarcinoma. *MYBL2* High lung adenocarcinomas feature inactivating alterations of *TP53* and *RB1* tumor suppressors, defects in TC-NER, and evidence of chronic replication stress (**Figure S3B**, **Figures 4C**, **6A, B, D**). As a consequence, *MYBL2* High tumors upregulate pathways that sense replication fork stress, mediate intra-S DNA damage checkpoints, and drive error-prone MMEJ repair. ChIP-seq data indicated that the MYBL2:LIN54 transcriptional complex directly upregulated genes that protect replication forks (RAD51, CHEK1, TOPBP1), promote error-prone MMEJ repair (POLQ, FEN1, PARP2), and mediate HJ-rejection (BLM, RMI2, MSH2, MSH6) (**Figure 5**). The notion that MYBL2-driven transcriptional programs are responsible for initiating and sustaining these DNA damage responses is supported by transcriptomic, COSMIC, and proteomic data (**Figures 5**, **6**).

It has long been recognized that defects in double-strand DNA break repair give rise to genomic instability and disease progression. However, the molecular programs promoting genomic instability in tumors lacking mutations in HR effectors, such as BRCA1/2, have remained elusive. In this study, we demonstrate that *MYBL2* High lung adenocarcinomas upregulate transcriptional programs that coordinate replication stress responses and POLQ-mediated error-prone repair despite containing BRCA proficient pathways (**Figure 5**, **Figure S4**). This finding builds on recent evidence that tumor cells preferentially drive error-prone repair at sites of replication stress and fork collapse (45). In addition to upregulating error-prone repair pathways, *MYBL2* High tumors actively antagonized HR repair by promoting HJ-rejection. HJ-rejection is recognized as an important repair process that prevents HR when mismatched nucleotides are present in either the homologous sequences in the sister chromatid or in the invading DNA sequence to be repaired (40, 42). In normal cells, BLM-RMI-mediated HJ-rejection antagonizes HR and allows cells to repair mismatches prior to undergoing recombination repair (40). MMR is carried out by a tetrameric complex that scans and identifies mismatched nucleotides (MSH2:MSH6) and facilitates repair of the mismatched nucleotides (MLH1:PMS2 or MLH1:MLH3) (40). Evidence provided indicates that *MYBL2* High lung adenocarcinomas overexpress MSH2 and MSH6 but lack MLH1 and MLH3 repair effectors (**Figures 5E**, **4C**). Together, this explains a mechanism by which tumors can detect (MSH2:MSH6) but cannot effectively repair mismatched nucleotides (MLH1:MLH3). This imbalance of MMR proteins drives HJ-rejection, antagonizes faithful HR, and promotes MMEJ repair (**Figure 5E**). Our data supports a mechanism wherein *MYBL2* High tumors with defective MMR suppress faithful HR through HJ-rejection and drive MMEJ repair. Evidence that defective MMR pathways suppress HR and favor MMEJ repair is supported by elevated COSMIC signature 3, which quantifies mutations associated with elevated large (>3 bp) insertions and deletions with overlapping microhomology at breakpoint junctions (*p* = 5.1e–3, **Figure 6D**) (43). While *MYBL2* High lung adenocarcinomas show evidence of elevated MMEJ, it is worth pointing out that these are conservative estimates observed at the genome-wide level and the actual level of genomic alterations facilitated by dysregulated MMEJ would be predicted to be even higher.

Knowing the importance of MYBL2 in disease progression, it is important to assess whether MYBL2 status predicts poor responses to current standard-of-care therapies such as surgical resection, irradiation, and/or systemic chemotherapy regimens. Currently, it is difficult to address these questions due to the lack of large patient cohorts that have detailed RNA-seq, treatment, and longitudinal follow-up data. In the next several years these questions will be addressed as collaborations, such as the ORIEN consortium, accrue large patient cohorts with detailed omics and treatment data required to make meaningful outcome predictions. In the meantime, establishing methods for identifying *MYBL2* High tumors in the clinic are crucial. To begin to address this issue, we have developed an RNA-based profiling panel and a candidate IHC panel to help identify *MYBL2* High disease (**Figures 8A, B**). While IHC has been successfully used to detect phospho-specific MYBL2 in human carcinomas, a more feasible approach would be to employ IHC panels detecting MYBL2 and MYBL2-regulated targets such as FOXM1, CHK1, and RAD51 (**Figures 6B**, **8B**) (46). Pending extensive validation, use of these or similar technologies will allow for identification of *MYBL2* High tumors at diagnosis and initiation of appropriate treatment regimens.

Moving forward, our results have important implications for utilizing CHK1-targeted therapies for the future treatment of *MYBL2* High lung adenocarcinoma. Consistent with previous findings in other carcinomas, *MYBL2* High tumors frequently carry inactivating alterations in *TP53* and *RB1* tumor suppressor genes (**Figure S3**) (36). Combined *TP53* and *RB1* inactivation impairs cellular capacity for G1/S cell cycle arrest. The loss of G1/S cell cycle arrest, combined with defects in NER and MMR pathways, produce chronic replication stress and induce a CHK1-dependent intra-S phase cell cycle arrest. Our observation that treatment naïve *MYBL2* High tumors overexpress active phoshpo-CHK1 protein supports the investigation of CHK1 inhibitors as a first line therapy for *MYBL2* High disease (**Figure 6B**). Consistent with *MYBL2* High lung adenocarcinomas upregulating CHK1-dependent checkpoint repair pathways, we find that cell lines with increased MYBL2 expression concomitantly upregulate CHK1 protein expression (**Figure 7A**). Importantly, *MYBL2* High cell lines are sensitive to prexasertib treatment as a single agent across multiple cell lines at nanomolar doses (**Figure 7D**). While it remains to be determined why prexasertib outperforms MK-8776 and rabusertib (**Figure 7B**), perhaps the best explanation is that, unlike these other inhibitors, prexasertib is a potent CHK1 and CHK2 inhibitor (47). Thus, inhibitors such as prexasertib, which effectively target both CHK1 and CHK2 would be predicted to be effective therapeutic options for *MYBL2* High lung adenocarcinomas (47). Additional support for prexasertib as a clinical trial agent for *MYBL2* High lung adenocarcinomas is provided by a Phase 2 clinical trial recently carried out in high grade serous ovarian cancer (HGSOC) (48). Much like *MYBL2* High lung adenocarcinoma, hallmarks of high grade serous ovarian cancer include TP53 mutations, replication stress, defective DNA repair, and widespread genomic instability (48, 49). Lee and colleagues report that prexasertib was well tolerated and produced significant antitumor responses in patients with recurrent *BRCA1/2* wildtype HGSOC. Importantly, unlike other CHK inhibitors, prexasertib administration did not induce cardiotoxicity (48). Since elevated MYBL2 is commonly observed in carcinomas with HR defects and combined *TP53* and *RB1* genetic alterations, our study supports the use of CHK inhibitors for other carcinomas, including small cell lung cancer. This idea is supported by preclinical trials using small cell lung cancer models (50). Additionally, our study demonstrates that *MYBL2* High tumors overexpress transcripts encoding two rate-limiting enzymes, RAD51 and POLQ. Because both RAD51 and POLQ have been shown to be key drivers of genomic instability, small molecule inhibitors to these proteins have been developed (16, 51–53). It will be important to examine the efficacy of CHK1 inhibitors in combination with either RAD51 or POLQ small molecules when treating *MYBL2* High lung adenocarcinomas. These two combinations are particularly intriguing due to the potential for direct inhibition of replication fork protection (RAD51) or MMEJ repair (POLQ). Given the increased likelihood for disease recurrence with *MYBL2* High tumors, promising new inhibitors need to be explored following disease relapse or in combination with current standard-of-care regimens (**Figures 2B, D**). Finally, it will be interesting to explore how targeted small molecule inhibitors described above could be combined with immune checkpoint blockade. This point is highly relevant since efficient dampening of the DNA damage response has been shown to increase checkpoint blockade success in various solid tumors (54).

Collectively, our study highlights the importance of MYBL2 in coordinating replication stress responses and error-prone repair in lung adenocarcinomas with proficient HR pathways. *MYBL2* High disease not only constitutes one of the most aggressive subtypes of lung adenocarcinoma but it also encompasses a large cohort of patients (~21% of all lung adenocarcinoma). Based on current cancer statistics, *MYBL2* High lung adenocarcinoma is estimated to represent 21,067 new cases this year alone. Therefore, the identification and development of novel therapeutic strategies, including CHK1/CHK2 inhibitors, for the treatment of *MYBL2* High disease will provide significant clinical benefit.

## Data Availability Statement

The data analyzed in this study is subject to the following licenses/restrictions: Access to ORIEN data is controlled by M2Gen and the ORIEN consortium. Requests to access these datasets should be directed to https://www.oriencancer.org/request-an-account. Publicly available datasets were analyzed in this study. These data can be found here: TCGA Firehose Legacy data can be found in cBioPortal (https://www.cbioportal.org/) (20). Genomic data and DNA repair metrics are available from Knijenburg et al. (20, Supplementary file “TCGA_DDR_Data_Resources.xlsx”). ChIP sequencing data can be found in the Gene Expression Omnibus (GEO) (https://www.ncbi.nlm.nih.gov/geo/) (MYBL2, GSM1010876; H3K27Ac, GSM733743; H3K4me3, GSM733737). COSMIC signature data can be found in the mSignatureDB database (http://tardis.cgu.edu.tw/msignaturedb/).

## Author Contributions

BM conceptualized the study, contributed to the investigation, formal analysis, writing the original draft, and writing, reviewing, and editing the manuscript. NW contributed to the formal analysis and wrote, reviewed, and edited the manuscript. PG wrote, reviewed, and edited the manuscript. PS wrote, reviewed, and edited the manuscript. RH, RG, WA, TV, SA, TW, and VC provided the resources, and wrote, reviewed, and edited the manuscript. DJ and DA wrote, reviewed, and edited the manuscript. MM conceptualized the study, acquired funding, supervised the study, wrote the original draft, and wrote, reviewed, and edited the manuscript. All authors contributed to the article and approved the submitted version.

## Funding

This work was supported by the National Cancer Institute (NCI R01 CA192399 to MM, NCI T32 CA009109-42 and NCI T32 CA009109-43 to BM, NCI R01 CA217169 and NCI R01 CA234617 to DJ, and P30 CA0044579-26 to NW) and the National Institutes of Health (NIH R01 GN118798 to PS and NIH R01 GM111911 to PG). Patient consent, specimen procurement, specimen processing, data abstraction, and access to molecular and clinical data were supported in part by the UVA Cancer Center Support Grant, P30CA044579. Funding sources listed were not involved in the design of this study, the analysis or interpretation of the data, the writing of this manuscript, or the decision to submit for publication.

## Conflict of Interest

RG has received research support from Pfizer, Merck, Takeda, Jounce Therapeutics, Helsinn, Bristol Myers Squibb, and Celgene as well as personal fees from AstraZeneca, Pfizer, Merck, Bristol Myers Squibb, and Ariad. RH has received research support from Merck, AstraZeneca, Mirati Therapeutics, and Abbvie as well as personal fees from Pfizer and Takeda. SA has received research funding from AstraZeneca, Amgen, Genentech, Merck Sharp & Dohme, Nektar Therapeutics, Exelixis Inc., and Kura Oncology. DJ serves as a senior medical advisor for Diffusion Pharmaceuticals and as a consultant for Merck and AstraZeneca. BM and MM have a provisional patent Serial No. 62/928,018.

The remaining authors declare that the research was conducted in the absence of any commercial or financial relationships that could be construed as a potential conflict of interest.

## Supplementary Material

Supplementary Data Sheet 1 TCGA RAD51 High & Low DE RNA-seq DE RPPA. Contains all significant (*q* < 0.05) 1) differentially expressed genes and 2) proteins when comparing TCGA *RAD51* High and Low tumors. Differentially expressed genes (RNA-seq) and differentially expressed proteins (RPPA) are presented as two separate Excel sheets.

Supplementary Data Sheet 2 TCGA MYBL2 High & Low DE RNAseq DE RPPA. Contains all significant (*q* < 0.05) 1) differentially expressed genes and 2) proteins when comparing TCGA *MYBL2* High and *MYBL2* Low tumors. Differentially expressed genes (RNA-seq) and differentially expressed proteins (RPPA) are presented as two separate Excel sheets.

Supplementary Data Sheet 3 MYBL2 ChIP-seq Analysis. ChIP-seq analysis of DNA damage response genes. MYBL2 ChIP-seq peaks found at gene promoters are listed by enrichment peak ID and are accompanied by corresponding genomic sequence.

Supplementary Data Sheet 4 MYBL2 High Panel Analysis. OS, DFS, and log-rank *p*-values for **Figure 8A** panel genes. Each gene tracks independently with poor OS and/or DFS outcomes when assessed using TCGA data.

Supplementary Data Sheet 5 TCGA ORIEN Clinical Data Tables. Full clinical data for TCGA and ORIEN cohorts presented in this study. Data for TCGA and ORIEN cohorts are presented on two separate Excel sheets.

Supplementary Table 1 COSMIC Signature Analysis. Table depicting average mutational signature contribution for all 30 COSMIC signatures across TCGA *MYBL2* High and Low cohorts.

## Abbreviations

NHEJ, Non-homologous end joining; HR, Homologous recombination; NSCLC, Non-small cell lung cancer; MYBL2, MYB proto-oncogene like 2; TCGA, The Cancer Genome Atlas; ORIEN, Oncology Research Information Exchange Network; FDR, False discovery rate; OS, Overall survival; DFS, Disease-free survival; MMS, Median month survival; RNA-seq, RNA sequencing; GSEA, Gene set enrichment analysis; COSMIC, Catalogue of Somatic Mutations in Cancer; ChIP-seq, Chromatin Immunoprecipitation sequencing; Combined HRD, Combined homologous recombination deficiency; NtAI, Telomeric allelic imbalances; LST, Large scale transition; LOH, Loss of heterozygosity; RPS, Repair proficiency score; DDR, DNA damage response; FA, Fanconi anemia; TLS, Translesion synthesis; DR, Direct repair; BER, Base excision repair; NER, Nucleotide excision repair; MMR, Mismatch repair; MMEJ, Microhomology-mediated end joining; HJ-rejection, Holiday junction rejection; TC-NER, Transcription-coupled nucleotide excision repair; IHC, Immunohistochemistry.
