# Supplementary figures and images for: MYBL2-Driven Transcriptional Programs Link Replication Stress and Error-prone DNA Repair With Genomic Instability in Lung Adenocarcinoma
Source: Front Oncol. 2021 Jan 8;10:585551. doi: 10.3389/fonc.2020.585551 (PMC7821388; doi:10.3389/fonc.2020.585551)

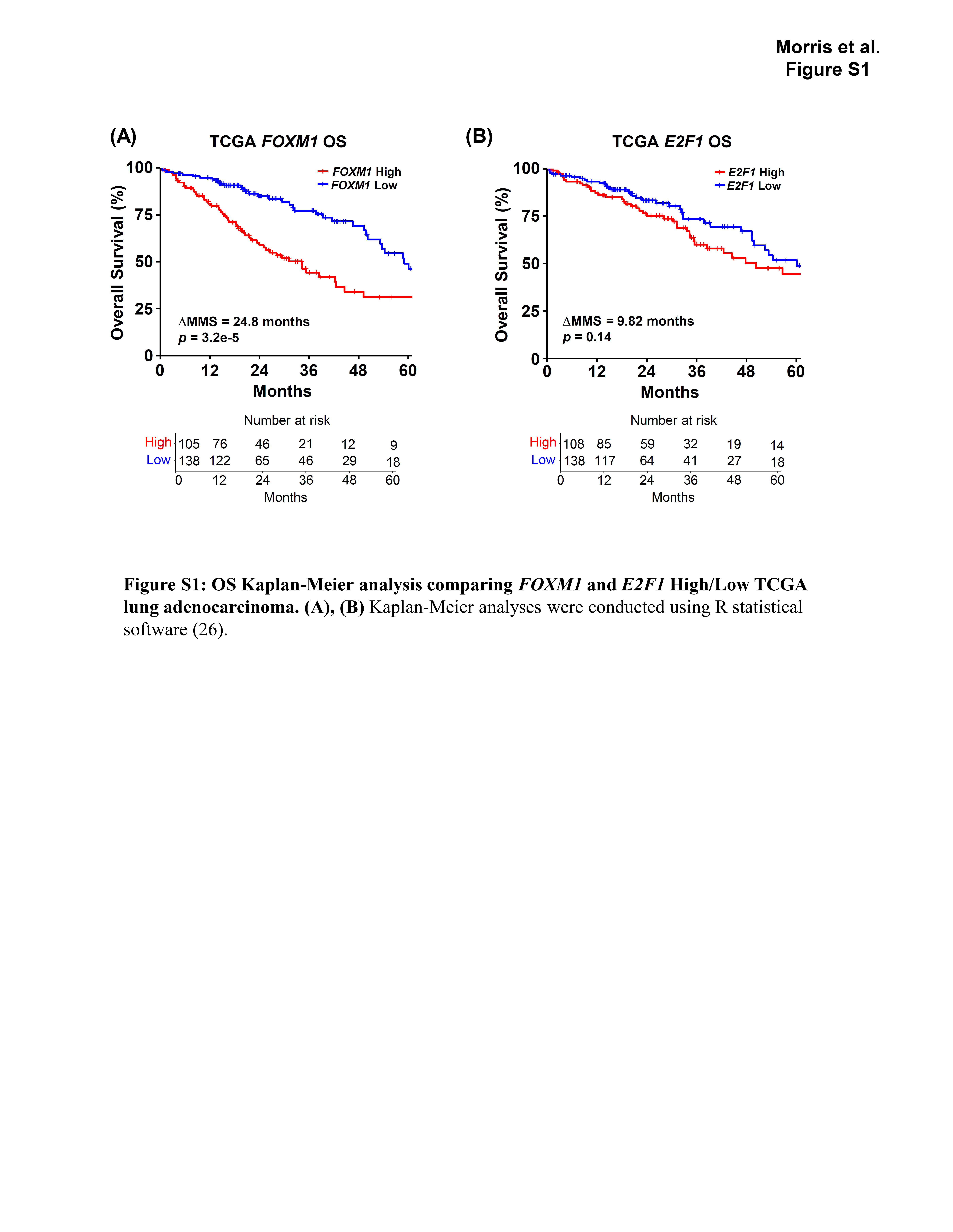

Supplement: Supplementary Data Sheet 1 — TCGA RAD51 High & Low DE RNA-seq DE RPPA. Contains all significant (q < 0.05) 1) differentially expressed genes and 2) proteins when comparing TCGA RAD51 High and Low tumors. Differentially expressed genes (RNA-seq) and differentially expressed proteins (RPPA) are presented as two separate Excel sheets. [file Image_1.jpeg]

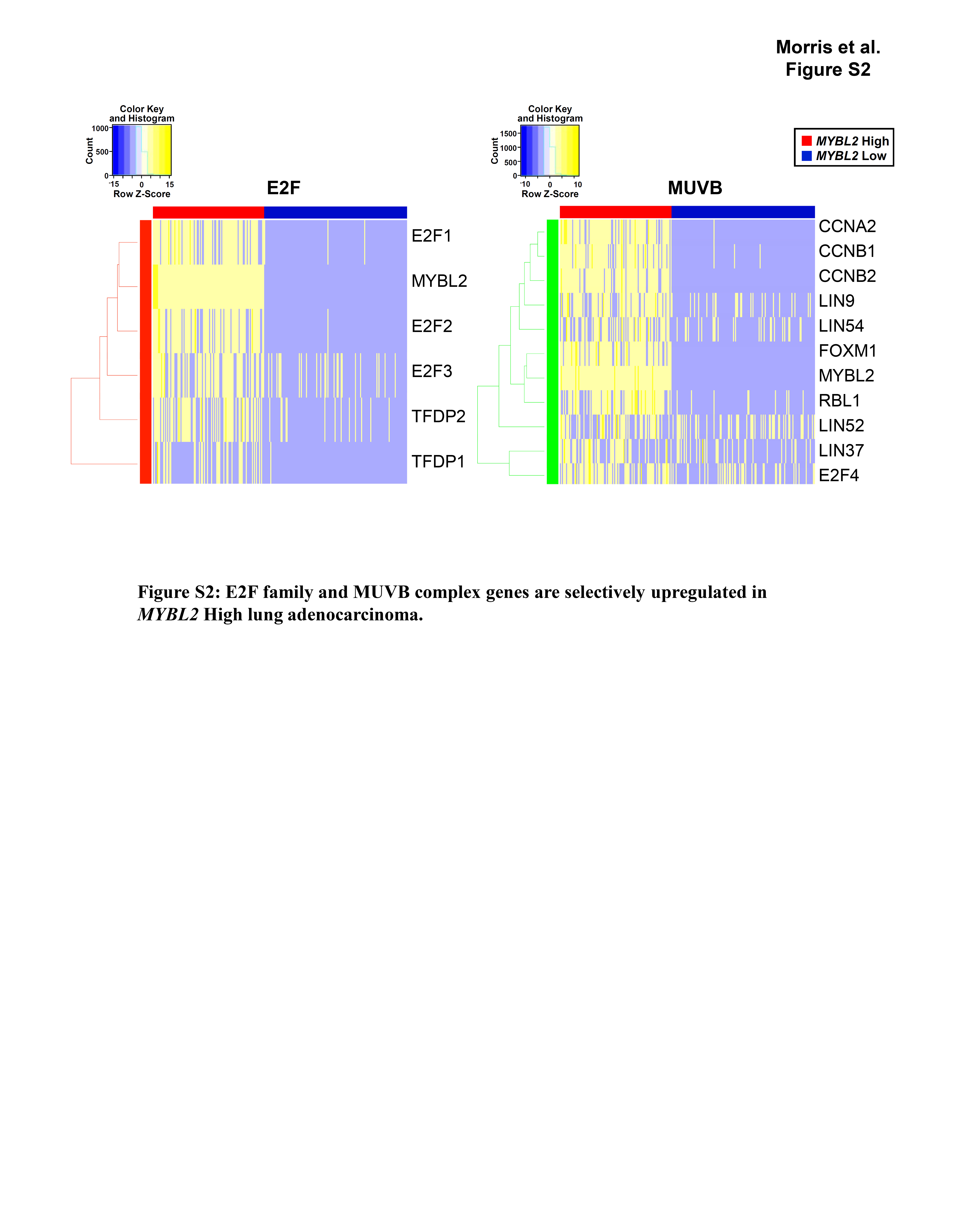

Supplement: Supplementary Data Sheet 2 — TCGA MYBL2 High & Low DE RNAseq DE RPPA. Contains all significant (q < 0.05) 1) differentially expressed genes and 2) proteins when comparing TCGA MYBL2 High and MYBL2 Low tumors. Differentially expressed genes (RNA-seq) and differentially expressed proteins (RPPA) are presented as two separate Excel sheets. [file Image_2.jpeg]

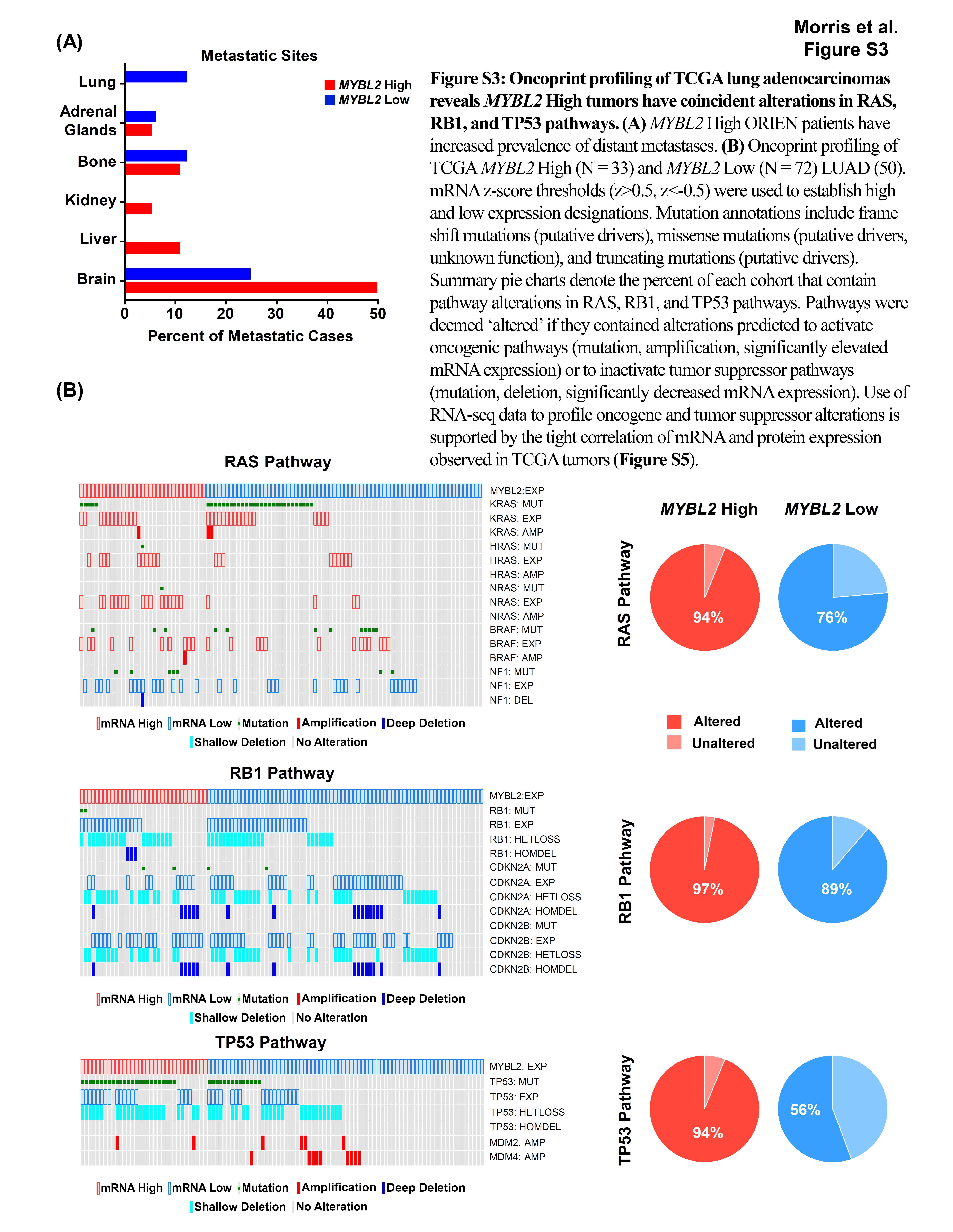

Supplement: Supplementary Data Sheet 3 — MYBL2 ChIP-seq Analysis. ChIP-seq analysis of DNA damage response genes. MYBL2 ChIP-seq peaks found at gene promoters are listed by enrichment peak ID and are accompanied by corresponding genomic sequence. [file Image_3.jpeg]

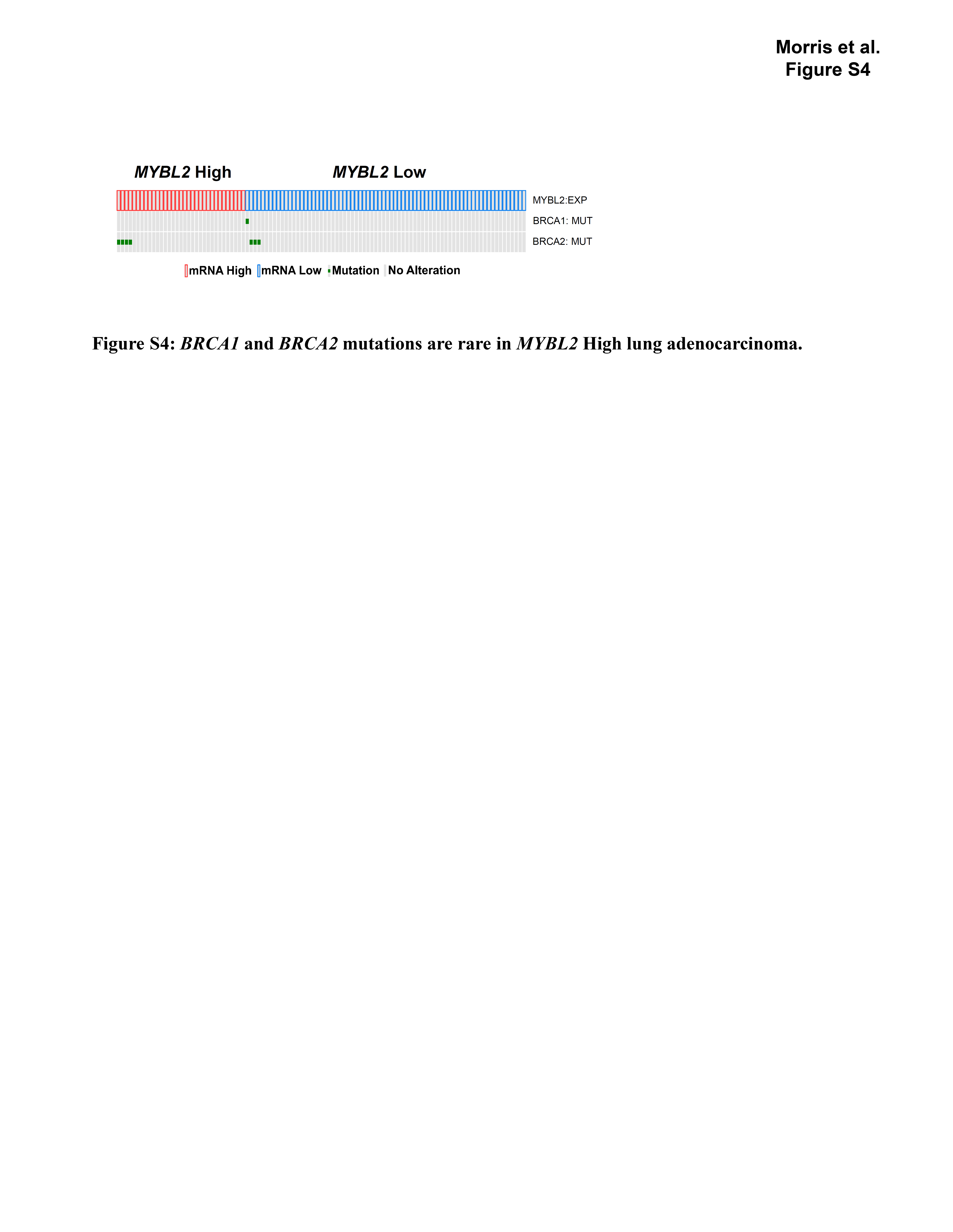

Supplement: Supplementary Data Sheet 4 — MYBL2 High Panel Analysis. OS, DFS, and log-rank p-values for Figure 8A panel genes. Each gene tracks independently with poor OS and/or DFS outcomes when assessed using TCGA data. [file Image_4.jpeg]

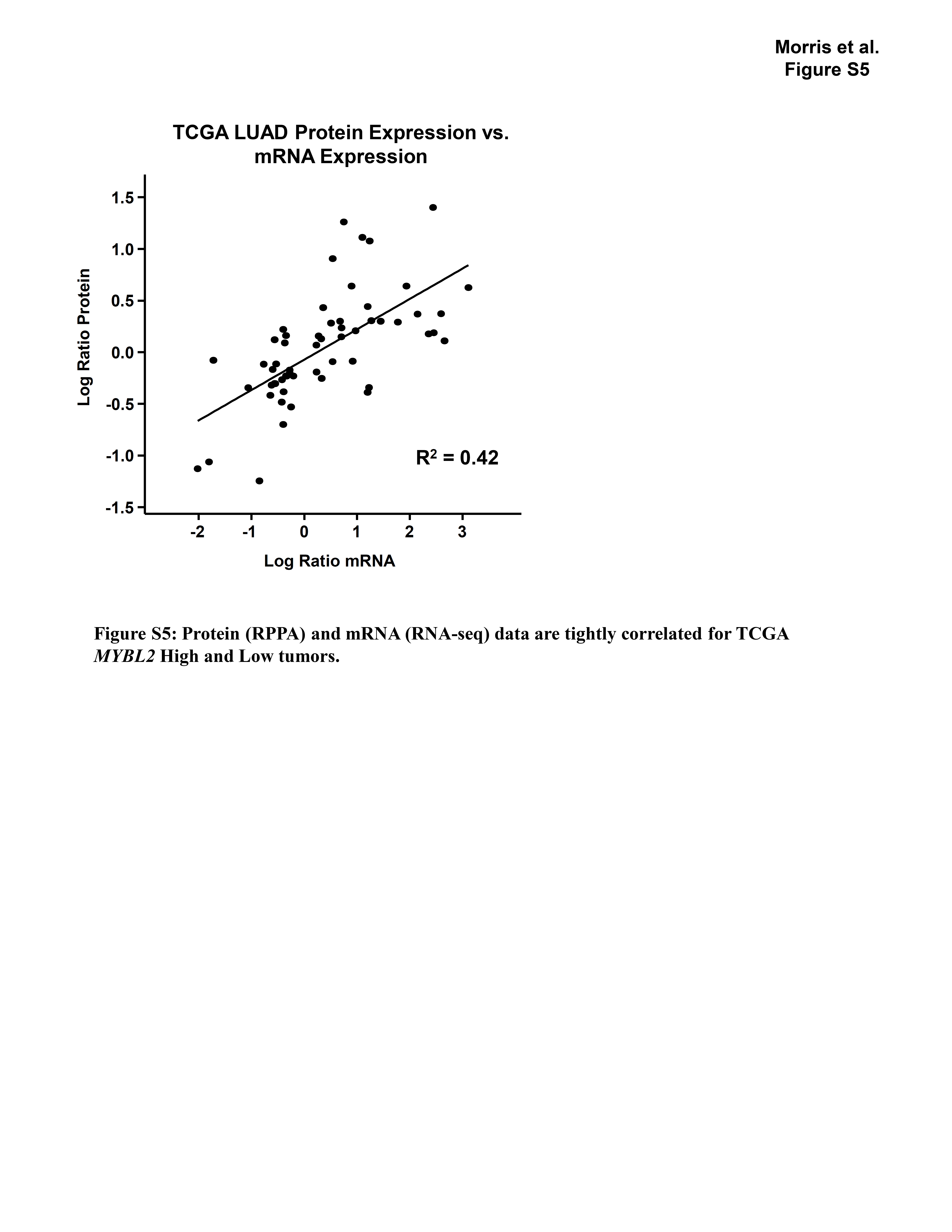

Supplement: Supplementary Data Sheet 5 — TCGA ORIEN Clinical Data Tables. Full clinical data for TCGA and ORIEN cohorts presented in this study. Data for TCGA and ORIEN cohorts are presented on two separate Excel sheets. [file Image_5.jpeg]
